# Antenatal care use and its determinants among migrant women during the first delivery: a nation-wide cross-sectional study in China

**DOI:** 10.1186/s12884-019-2520-3

**Published:** 2019-10-15

**Authors:** Xue Tang, Lulu Ding, Yuejing Feng, Yi Wang, Chengchao Zhou

**Affiliations:** School of Public Health, Shandong University; NHC Key Laboratory of Health Economics and Policy Research (Shandong University), 44 Wen-hua-xi Road, Jinan, 250012 Shandong China

**Keywords:** Antenatal care, Times, Determinants, Migrant maternal, First delivery

## Abstract

**Background:**

Reasonable use of antenatal care (ANC) services by pregnant women played a crucial role in ensuring maternal and child safety and reducing the risk of complications, disability, and death in mothers and their infants. This study aimed to investigate the ANC use, and to explore the factors associated with ANC use among migrant women during the first delivery in China.

**Methods:**

This study used the data of National Health and Family Planning Commission of People Republic of China in 2014. A total of 1505 migrant primiparous women were included in our current analysis. Frequencies and proportions were used to describe the data. Chi-square tests and multivariate binary logistic regression models were performed to explore the determinants that affect the number of times migrant women used ANC during their first delivery.

**Results:**

Of the 1505 participants, 279 (18.54%) women received the ANC less than 5 times, and 1226 (81.46%) women used the ANC at least 5 times during the first delivery. The multivariate logistic regression model showed that migrant primiparous women with college and above education(*P* < 0.05;OR = 2.57;95%CI = 1.19–5.55), from the households with higher monthly income (*P* < 0.01;OR = 2.01;95%CI = 1.30–3.13), covered by maternity insurance(*P* < 0.01;OR = 2.01;95%CI = 1.28–3.18), with maternal health records (*P* < 0.001;OR = 2.44;95%CI = 1.61–3.69), migrating across county (*P* < 0.05;OR = 2.57;95%CI = 1.14–5.81), having migration experience before pregnancy(*P* < 0.05;OR = 1.37;95%CI = 1.03–1.81) were more likely to use ANC for at least five times.

**Conclusions:**

This study demonstrated that there were still some migrant maternal women (18.54%) who attended the ANC less than 5 times. Targeted policies should be developed to improve the utilization of ANC among migrant pregnant women.

## Background

With the rapid development of social economy and urbanization, the size of China’s migrant population continues to grow [1–3]. According to *the Report of Migrant Population Service Center, National Health Commission, People’s Republic of China (PRC)*, the number of migrant population has reached 244 million by the end of 2017 [4]. At the same time, with the gradual emergence of the “family migration style (families migrate together)”, more and more women migrated with their husbands. Migrant childbearing women account for about one-fourth of the national married women [5, 6]. As a consequence, the number of migrant women who use maternal basic public health services in the inflow areas is also on the rapid rise. According to the National Basic Public Health Service, nearly 70% of migrant women chose to use antenatal care (ANC) and delivery services in the inflow areas [7].

According to the “Chinese citizens’ health literacy - basic knowledge and skills” issued by the former Ministry of Health, PRC, pregnant women should take the initiative to accept pre-marital and pre-pregnancy care, and should use at least five times of antenatal care during pregnancy [8]. In addition, according to National Basic Public Health Service Project, pregnant women may go to the community health center or township health center to enjoy five times of free ANC, including pregnant women health assessment (medical history, family history, etc.), general physical examination, gynecological examination, blood routine, routine urine, liver function, renal function, and hepatitis B test [9]. National Basic Public Health Service Project covers not only the resident population, but also the migrant population. The migrant pregnant woman can enjoy the service fairly.

Reasonable use of ANC services for the recommended number of times according to the national guideline for the pregnant women plays a crucial role in ensuring maternal and child safety and reducing the risk of comorbidities, disability, and death in mothers and their infants [3, 10, 11]. Only those maternal women who used prenatal care were able to get medical follow-up, advice and information when needed [11]. ANC not only affected clinical pregnancy, but also had a psychological effect, preparing for the mother to give birth [12, 13]. However, the ANC service use was poorer among migrant pregnant women compared to the registered local pregnant women [14]. Therefore, we would explore the determinants of the ANC use among the migrant women, and provide some suggestions for further improving ANC use for migrant pregnant women.

Previous studies have explored determinants of ANC use among pregnant women [15–17]. A study in Nepal found that factors including age, education, and household wealth were associated with both the timing of prenatal care and the number of ANC care visits [18]. Another study in Turkey indicated that educational attainment, parity level, health insurance coverage, ethnicity, household wealth, and geographic region were statistically significant factors that affect maternal health care [19]. A study by Chen X et al. showed that education level, household income were associated with prenatal examination use in Hubei, China [20]. However, to date, few studies have explored the determinants of ANC use among migrant pregnant women, and no studies have focused on the ANC use at first pregnancy. The experience of maternal health care use during the first pregnancy was extremely important for the pregnant women. This study aims to explore the determinants of ANC use during the first pregnancy among the migrant women in China. To do so, we have two specific objectives: to identify the number of times migrant women used ANC during their first pregnancy, and to explore the determinants of the ANC use among the migrant women in China.

## Methods

### Data source

The data of this study were derived from the 2014 National Internal Migrant Population Dynamic Monitoring Survey (NIMPDMS), conducted by the National Health and Family Planning Commission, PRC. The survey used a multi-stage, stratified sampling method, to select participants in 31 provinces, autonomous regions, municipalities, and Xinjiang Production and Construction Corps in China. More details about data collection methods were reported in our previous studies [21].

In this study, the migrants refer to those who move from Hukou region to another place and live for more than one month. The inclusion criteria of the respondents were: 1) the women with the age range of 15 to 49 years old; 2) those were under migrant status during the first pregnancy; 3) those were primiparas. A total of 4417 migrant women met the first criterion, of whom 1927 respondents could not remember the times of ANC during the first pregnancy, 159 respondents did not have information on key variables (i.e., ANC use), and 345 respondents were multiparous women. Finally, 1505 eligible migrant women were included in the analysis. Some more details for the selecting process of the respondents were presented in the Appendix Fig. 1. Our team has acquired the license to access the data used in this study.

### Variables and measures

#### Dependent variable

The dependent variable was the times of ANC use during the first pregnancy, and the question was “How many times have you used ANC during your first pregnancy?” The answer was originally quantitative, according to the “Chinese citizens’ health literacy - basic knowledge and skills” issued by the former Ministry of Health, China, pregnant women should attend at least five ANC during pregnancy, so the answers were divided into two categories: < 5 times, and ≥ 5 times.

#### Independent variable

The independent variables consisted of four sections: demographic characteristics, economic characteristics, reproductive health knowledge, and migration characteristics. Demographic characteristics included ethnic groups (Hans versus Minority), age at first delivery (≤24, > 24 years), education (primary school and below, junior school, high school, college and above), Hukou (rural, urban). Economic characteristics included household monthly income (Q1, Q2, Q3 and Q4), quartiles of the household monthly income, quartile 1(Q1) was the lowest income and quartile 4(Q4) was the highest income, kinds of insurance (0,1, ≥2), maternity insurance (no, yes). Here, household monthly income is the total monthly income of all family members. Maternity insurance is a social insurance system that provides medical services, maternity benefits. Reproductive health knowledge included maternal health education (no, yes), maternal health records (no, yes). Maternal health education includes reproduction education and contraception education, which is provided to pregnant women. Maternal health record is a record to keep maternal and infant physical health examination throughout pregnancy stages, childbirth, puerperium and 42 days postpartum. Migration characteristics included migration range (across province, across prefectural city, across county), migration reasons (working or doing business, accompanying, others), migration experience before pregnancy (no, yes).

### Statistical analysis

The data were described using frequencies and proportions when appropriate. Chi-square tests was used to analyze the potential factors affecting the times of ANC use during first pregnancy. Four step-by-step forward multiple binary logistic regression models were established to identify variables associated with the times of ANC use during the first pregnancy. The *P*-value < 0.05 was considered statistically significant in this study. Sampling weights were included in all analyses.

## Results

### Respondents’ sociodemographic characteristics

Table 1 showed the characteristics of 1505 migrant women included in this study. Of the women, the mean times of ANC was 7.45, and the standard deviation was 3.56, with a range from 0 to 42 times. 279(18.54%) women used ANC less than 5 times, and 1226 (81.46%) women used at least 5 times As for WHO recommendation (8 or more ANC visits during the pregnancy), 722(47.97%) migrant women used ANC at least 8 times during their first delivery. With respect to education level, 3.32% of the participants were primary school and below, 50.70% received junior school education, 26.91% received high school education, and 19.07% were college and above. 83.79% of the migrant women were from rural areas. 416(27.64%) women had maternity insurance, but 162(10.76%) women did not have any kind of insurance. In total, 1176(78.14%) women migrated to work and do business and more than half of the participants had migration experience before the pregnancy (66.45%).

**Table 1:** Basic information of the migrant women during the first delivery in China, 2014

Characteristics	Number	Percentage%
Times of ANC Visits		
< 5	279	18.54
≥ 5	1226	81.46
Ethnic groups		
Minority	49	3.26
Hans	1456	96.74
Age at first delivery (years)		
≤ 24	678	45.05
> 24	827	54.95
Education		
Primary school and below	50	3.32
Junior school	763	50.70
High school	405	26.91
College and above	287	19.07
Hukou^a^		
Rural	1261	83.79
Urban	244	16.21
Household monthly income ^b^		
Q1	304	20.20
Q2	488	32.43
Q3	351	23.32
Q4	362	24.05
Kinds of insurances		
0	162	10.76
1	727	48.31
≥ 2	616	40.93
Maternity insurance ^c^		
No	1089	72.36
Yes	416	27.64
Maternal health education ^d^		
No	534	35.48
Yes	971	64.52
Maternal health records ^e^		
No	124	8.24
Yes	1381	91.76
Migration range		
Across province	760	50.50
Across prefectural city	666	4.25
Across county	79	5.25
Migration reasons		
Working or doing business	1176	78.14
Accompanying	306	20.33
Others	23	1.53
Migration experience before pregnancy		
No	505	33.55
Yes	1000	66.45

^a^ Hukou is the household registration system in China. A Hukou record officially identifies a person as a resident of a particular place. Residents of China are mainly categorized into two types, rural Hukou and urban Hukou

^b^ Quartiles of the household monthly income, and Quartile 1(Q1) is the poorest and Quartile 4(Q4) is the richest

^c^ Maternity insurance is a social insurance system that provides medical services, maternity benefits and maternity leave by the state and society when women workers who are pregnant and giving birth temporarily interrupt their labor

^d^ Maternal health education included reproduction education and contraception education

^e^ Maternal health records is a record of maternal and infant health examination throughout their pregnancy, childbirth, puerperium and 42 days postpartum

### Factors associated with times of ANC use among migrant women during the first delivery

Table 2 presented the results of the univariate analysis for the ANC use. Age at first delivery, education, Hukou, household monthly income, kinds of insurances, maternity insurance, maternal health records within 12 weeks of pregnancy, migration range, migration experience before pregnancy were associated with the ANC use among migrant women during the first delivery in China(*P* < 0.05). A stepwise regression was used to explore the determinants of the ANC use among migrant women during the first delivery in Table 3. Model 1 reflected the associations of sociodemographic characteristics and ANC use among migrant women. The results revealed that the age at first delivery and education level were associated with ANC use. After the inclusion of economic factors in Model 2, age and education were still associated with the use of ANC among migrant women, and household monthly income and maternity insurance status were associated with use of ANC. In Model 3, we included the factors of reproductive health knowledge, and the results showed that factors including age at first delivery, education, household monthly income, and maternity insurance were still significant associated with use of ANC. Women who had maternal health records within 12 weeks of pregnancy (*P* < 0.001) were 2.48 times more likely to use ANC than women who had no records. When adding the variables of migration characteristics in Model 4, we found that, besides the factors we identified in Model 3, migration range and migration experience before the pregnancy were associated with use of ANC. The migrant women who migrated across prefectural city (*P* < 0.01) and across county (*P* < 0.05), were 1.52 times and 2.57 times more likely, respectively, to use the ANC than those migrated across province. Migrant women who had migration experience before pregnancy (*P* < 0.05) were 1.37 times more likely to use ANC at least 5 times than their counterpart. The Appendix Table 4 showed the result of Hosmer-Lemeshow tests of the four models, which indicated that the models we built were reasonable. Cox & Snell R^2^and Nagelkerke R^2^ of the models showed that the model 4 had the best goodness of fit.

**Table 2:** Univariate analysis of factors associated with times of ANC use among migrant women during the first delivery in china, 2014

Characteristics	Times of ANC visits(^−^x ± S)	Times of ANC visits		*P*-value	χ^2^
		< 5	≥5		
Ethnic groups				0.975	0.001
Minority	7.69 ± 3.59	9 (18.37)	40 (81.63)		
Hans	7.44 ± 3.56	270 (18.54)	1186 (81.46)		
Age at first delivery (years)				<0.001	12.304
≤ 24	6.86 ± 3.26	152 (22.42)	526 (77.58)		
> 24	7.94 ± 3.71	127 (15.36)	700 (84.64)		
Education				< 0.001	27.544
Primary school and below	6.04 ± 3.26	18 (36.00)	32 (64.00)		
Junior school	6.93 ± 3.31	166 (21.76)	597 (78.24)		
High school	7.59 ± 3.33	62 (15.31)	343 (84.69)		
College and above	8.87 ± 4.08	33 (11.50)	254 (88.50)		
Hukou^a^				0.043	4.087
Rural	7.21 ± 3.43	245 (19.43)	1016 (80.57)		
Urban	8.72 ± 3.90	34 (13.93)	210 (86.07)		
Household monthly income ^b^				< 0.001	18.276
Q1	6.79 ± 3.25	76 (25.00)	228 (75.00)		
Q2	7.13 ± 3.49	92 (18.85)	396 (81.15)		
Q3	7.24 ± 3.42	67 (19.09)	284 (80.91)		
Q4	8.65 ± 3.75	44 (12.15)	318 (87.85)		
Kinds of insurances				0.049	6.031
0	7.17 ± 3.27	33 (20.37)	129 (79.63)		
1	7.05 ± 3.32	150 (20.63)	577 (79.37)		
≥ 2	7.99 ± 3.82	96 (15.58)	520 (84.42)		
Maternity insurance ^c^				< 0.001	17.394
No	7.15 ± 3.49	230 (21.12)	859 (78.88)		
Yes	8.25 ± 3.60	49 (11.78)	367 (88.22)		
Maternal health education ^d^				0.071	3.251
No	7.21 ± 3.80	112 (20.97)	422 (79.03)		
Yes	7.58 ± 3.41	167 (17.20)	804 (82.80)		
Maternal health records ^e^				0.000	30.812
No	5.80 ± 4.59	46 (37.10)	78 (62.90)		
Yes	7.60 ± 3.41	233 (16.87)	1148 (83.13)		
Migration range				0.001	15.070
Across province	7.48 ± 4.07	168 (22.11)	592 (77.89)		
Across prefectural city	7.40 ± 2.94	104 (15.62)	562 (84.38)		
Across county	7.58 ± 2.97	7 (8.86)	72 (91.14)		
Migration reasons				0.564	1.145
Working or doing business	7.53 ± 3.66	224 (19.05)	952 (80.95)		
Accompanying	7.10 ± 3.06	52 (16.99)	254 (83.01)		
Others	8.13 ± 4.15	3 (13.04)	20 (86.96)		
Migration experience before pregnancy				0.015	5.963
No	7.25 ± 3.73	111 (21.98)	394 (78.02)		
Yes	7.55 ± 3.46	168 (16.80)	832 (83.20)		

^a^ Hukou is the household registration system in China. A Hukou record officially identifies a person as a resident of a particular place. Residents of China are mainly categorized into two types, rural Hukou and urban Hukou

^b^ Quartiles of the household monthly income, and Quartile 1(Q1) is the poorest and Quartile 4(Q4) is the richest

^c^ Maternity insurance is a social insurance system that provides medical services, maternity benefits and maternity leave by the state and society when women workers who are pregnant and giving birth temporarily interrupt their labor

^d^ Maternal health education included reproduction education and contraception education

^e^ Maternal health records is a record of maternal and infant health examination throughout their pregnancy, childbirth, puerperium and 42 days postpartum

**Table 3:** Multivariate analysis of factors associated with times of ANC use of migrant women during the first delivery in China, 2014

Characteristics	Model 1	Model 2	Model 3	Model 4
Ethnic groups (Minority^Ref^)				
Hans	0.99	0.87	0.72	0.62
	(0.47,2.11)	(0.41,1.88)	(0.33,1.59)	(0.28,1.37)
Age at first delivery (≤24year^Ref^)				
> 24	1.40*	1.39*	1.34*	1.26
	(1.06,1.84)	(1.05,1.84)	(1.01,1.79)	(0.94,1.68)
Education (Primary school and below^Ref^)				
Junior school	2.13*	2.08*	1.78	1.71
	(1.16,3.92)	(1.12,3.83)	(0.95,3.33)	(0.91,3.21)
High school	3.16***	2.99**	2.52**	2.43*
	(1.66,6.01)	(1.55,5.76)	(1.29,4.92)	(1.24,4.76)
College and above	4.20***	3.30**	2.67*	2.57*
	(2.03,8.69)	(1.56,6.99)	(1.24,5.75)	(1.19,5.55)
Hukou (Rural^Ref^) ^a^				
Urban	0.89	0.78	0.78	0.82
	(0.56,1.41)	(0.49,1.25)	(0.49,1.25)	(0.51,1.33)
Household monthly income(Q1^Ref^) ^b^				
Q2		1.43*	1.44*	1.48*
		(1.01,2.04)	(1.01,2.06)	(1.03,2.12)
Q3		1.32	1.26	1.30
		(0.90,1.93)	(0.86,1.86)	(0.88,1.92)
Q4		1.92**	1.87**	2.01**
		(1.25,2.95)	(1.21,2.89)	(1.30,3.13)
Kinds of insurances (0^Ref^)				
1		1.04	1.05	0.98
		(0.67,1.61)	(0.68,1.62)	(0.63,1.53)
≥ 2		0.75 (0.45,1.26)	0.72 (0.43,1.21)	0.73 (0.43,1.23)
Maternity insurance (No^Ref^) ^c^				
Yes		2.12**	2.18**	2.01**
		(1.35,3.33)	(1.39,3.44)	(1.28,3.18)
Maternal health education (No^Ref^) ^d^				
Yes			1.10	1.04
			(0.83,1.45)	(0.78,1.38)
Maternal health records (No^Ref^) ^e^				
Yes			2.48***	2.44***
			(1.64,3.74)	(1.61,3.69)
Migration range (Across Province^Ref^)				
Across prefectural city				1.52**
				(1.14,2.02)
Across county				2.57*
				(1.14,5.81)
Migration reasons				
(Working or doing business^Ref^)				
Accompanying				1.15
				(0.81,1.63)
Others				1.97
				(0.56,6.97)
Migration experience before pregnancy				
(N0^Ref^)				
Yes				1.37*
				(1.03,1.81)

^a^ Hukou is the household registration system in China. A Hukou record officially identifies a person as a resident of a particular place. Residents of China are mainly categorized into two types, rural Hukou and urban Hukou

^b^ Quartiles of the household monthly income, and Quartile 1(Q1) is the poorest and Quartile 4(Q4) is the richest

^c^ Maternity insurance is a social insurance system that provides medical services, maternity benefits and maternity leave by the state and society when women workers who are pregnant and giving birth temporarily interrupt their labor

^d^ Maternal health education included reproduction education and contraception education

^e^ Maternal health records is a record of maternal and infant health examination throughout their pregnancy, childbirth, puerperium and 42 days postpartum

^f^ *P-value< 0.05, ** *P*-value < 0.01, *** *P*-value < 0.001

## Discussion

Our results revealed that the average times of ANC among the migrant pregnant women during the first delivery was 7.45 ± 3.56, which was higher than the reported times of 6.2 ± 3.6 among the migrant pregnant women in Nanjing city in 2011 [22]. When taking the minimum threshold of five times into account, we found that 81.46% of the migrant women used at least five times of ANC at the first delivery, which was higher than the reported rate of 56.2% of migrant women in Nanjing city in 2011 [22], and 59.5% of migrant women in Beijing and Hangzhou city in 2009 [23]. There might be several explanations for this finding. First, China’s basic public health services have been implemented for about five years, and some achievements have already been made in maternal health. As a result, the ANC use have improved from the previous studies in 2009 and 2011 to this study in 2014. Second, compared to the migrant women who had experienced deliveries, the pregnant women themselves and their families tended to give more concerns during the pregnancy of the first delivery. It is worth noting that there were still 18.54% of migrant maternal who have had attended ANC less than 5 times, which needed to be improved in the future. The government should strengthen the publicity of the importance of ANC use so as to improve the ANC use among the migrant pregnant women.

Several previous studies found that the times of ANC was associated with increasing age [18, 24–26]. The current study also found that mother’s age at first delivery was associated with ANC use, and the mothers with older age were more likely to use ANC than those with younger age when giving birth to the first child. A study by Schimmel MS et al. indicated that the risk of delivery is higher in older women, and the incidence of perinatal complications and neonatal asphyxia is significantly higher than that in the younger women [27]. In order to ensure the safety of mothers and infants, regular prenatal examination during pregnancy has been informed to those older women, especially for those older ones at first delivery, of important clinical significance for early detection of abnormal conditions of mothers and infants, and then corresponding treatment measures were taken to prevent adverse maternal outcomes [28]. In addition, compared to those younger ones, those older primiparous women would be more careful about their first delivery, and then would use ANC more regularly. These might be the reasons why those older mothers at first delivery had higher ANC use.

Our study observed that women with higher education level used the ANC more often than those with lower education level, which was similar with some previous studies [19, 29]. There were several possible reasons for this observation. One explanation might be the effect of education on the awareness of the ANC use. According to the study by Streatfield et al. [30], highly educated women were more likely to be aware of the advantages of health care services, especially for those preventive health care. Thus, the educated women were more inclined to use ANC. Second, a study by Raghupathy et al. demonstrated that the women with higher education level tended to have higher autonomy than their counterparts. As a result, the women with higher autonomy would be more likely to use appropriate health care services to meet their needs, including ANC [31] .

The effect of household income on health service utilization had been demonstrated in some other populations in previous studies [10, 26, 32–35]. This study also found that the women at first delivery with higher household monthly income had higher possibility of using ANC than their counterparts. The women with high household income were more able to afford health care services, including ANC and related services. Our study also showed that maternity insurance played a significant role in the utilization of ANC services. In general, the women who have bought maternity insurance were those in good socioeconomic status [36], which we have discussed above. In addition, even though the ANC services were provided free for the pregnant women, some of the services (e.g., special checkup fees) were not covered in the free package, and maternity insurance would probably increase the ANC service accessibility for the pregnant women. Therefore, the government may provide more free maternal health care services to the poor and low-income migrant pregnant women, especially for those who did not have maternity insurance.

Migration range was found to be associated with ANC services use in migrant pregnant women. The further the distance of migration, the fewer times the migrant women utilized ANC services. In general, health policies, including maternal health policies, are similar in the same province or prefectural city, but are different across provinces. The women who migrated across county or prefectural city were close to their hometowns, and had a better understanding of local maternal health policies and health service systems. This finding implied for the policymakers in inflow areas to carry out more health educations about maternal health policies for those migrant pregnant women who were from other provinces.

### Limitations of the study

This study had some limitations. First, the data were self-reported, and the recall bias was inevitable. Second, there were also some other potential factors (e.g., the duration of stay in the inflow area, husband’s education, distance from the nearest community health center or township health center, etc.) that may affect the ANC use were not included in this study, which would be remedied in the follow-up study. Third, some of the respondents were excluded in this study which may result in potential bias.

## Conclusions

Migrant women with older age, with higher education level, with higher household income, who had maternity insurance, who established maternal health records, who migrated across county, and who had migration experience before pregnancy were more likely to use the ANC at least five times. The findings had several policy implications. First, comprehensive interventions should be conducted to further improve the ANC use among the migrant pregnant women. Second, the government should popularize the importance of ANC to pregnant migrant women and their families, especially for those who migrated across provinces. Future researches would focus on the impact evaluation of the potential inventions or policies on the improvement of ANC use among the migrant pregnant women in China.

## Figures and Tables

**Fig. 1:**
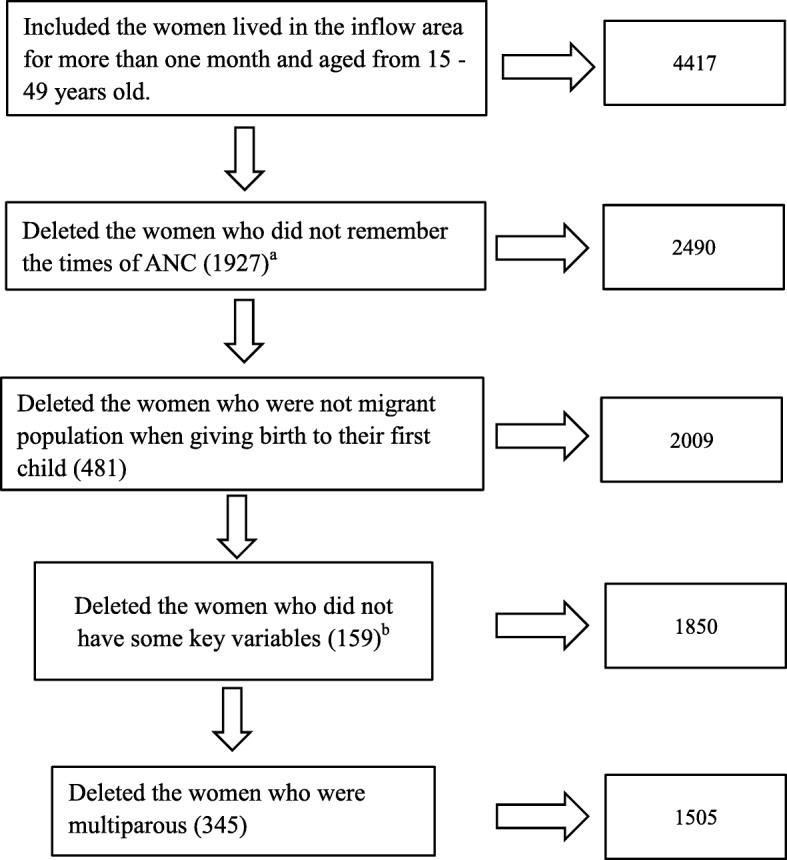
Sample inclusion and exclusion process. **a** The 1927 women could not remember the times of ANC when they were asked “How many times have you used ANC during your first pregnancy?”. **b** The 159 women did not have information on key variables

## Data Availability

The datasets analyzed during the current study are not publicly available due the datasets involve the confidentiality of data but are available from the corresponding author on reasonable request.

## Appendix

**Table 4:** The model test of the four models

Model	-2 Log likelihood	Cox & Snell R^2^	Nagelkerke R^2^	Hosmer-Lemeshow
Model 1	1410.517	0.021	0.035	*P* = 0.933
Model 2	1388.835	0.035	0.057	*P* = 0.896
Model 3	1370.353	0.047	0.077	*P* = 0.577
Model 4	1351.506	0.059	0.096	*P* = 0.682

## Abbreviations

ANC: Antenatal care
NIMPDMS: National Internal Migrant Population Dynamic Monitoring Survey
PRC: People’s Republic of China
